# Evaluation of Hemoglobin and Eosinophil Count in Patients Receiving Thrombolytic Treatment

**DOI:** 10.7759/cureus.64575

**Published:** 2024-07-15

**Authors:** Ramiz Yazıcı, Muhammed Güner, Ayşe F Basa Kalafat, Rabia B Tapkan, Hilmi Kaya, Bilal Yeniyurt, Salih Fettahoğlu, Utku M Kalafat, Efe D Bala, Serkan Doğan

**Affiliations:** 1 Emergency Medicine, İstanbul Kanuni Sultan Suleyman Training and Research Hospital, University of Health Sciences, İstanbul, TUR

**Keywords:** eosinophil, thrombolytic, ischemic stroke, immunomodulation, hemoglobin

## Abstract

Background and aim

Stroke ranks among the primary contributors to disability and mortality on a global scale. Recent advances in ischemic stroke pathophysiology emphasize the significant role of the immune system in both stroke-related damage and neuroprotection. This article investigates the relationship between hemoglobin level and white blood cell count.

Materials and methods

From January 1, 2019, to April 1, 2022, all patients aged 18 years and over who were diagnosed with acute ischemic stroke in the emergency department of Kanuni Sultan Süleyman Training and Research Hospital and treated with intravenous recombinant tissue plasminogen activator (r-tPA) within 4.5 hours of stroke onset were included in this cross-sectional retrospective study. Gender, age, onset of symptoms, complaints, National Institutes of Health Stroke Scale (NIHSS) score, stroke-affected area, as well as leukocyte, neutrophil, platelet, eosinophil, lymphocyte, and hemoglobin levels were recorded and compared between mortality and survivor groups.

Results

A total of 61 people, including 33 men and 28 women, were included in the study. Four patients died during follow-ups. The mean duration of symptoms upon admission was 86.23 ± 56.37 minutes. The mean NIHSS score of patients was found to be 9.16 ± 3.88 (minimum: 4, maximum: 18). There was a statistically significant positive correlation between age and symptom duration (p < 0.002, r: 0.391). A statistically significant negative correlation was found between eosinophil count and NIHSS score (p < 0.012, r: -0.321) and between eosinophil count and symptom duration (p < 0.042, r: -0.261). There was a negative correlation between hemoglobin levels and mortality (p < 0.013, r: -0.318). A statistically significant negative correlation was observed between the eosinophil-to-neutrophil ratio (ENR) and NIHSS score (p < 0.017, r: -0.305) as well as between ENR and symptom duration (p < 0.034, r: -0.271). Hemoglobin is a significant predictor of mortality in the logistic regression model (p < 0.05, CI: 0.253-0.942). For each one-unit increase in hemoglobin, the odds of mortality decrease by a factor of 0.488.

Conclusion

Certain blood cell types (neutrophils, eosinophils, and lymphocytes) play an active role in determining stroke prognosis. A detailed explanation of the role of leukocyte types lays the foundation for “immunomodulation,” which could be a promising novel treatment modality for future stroke patients.

## Introduction

Stroke ranks among the primary contributors to disability and mortality on a global scale. Ischemic stroke (IS), constituting more than two-thirds of all stroke types, carries a significant risk of enduring impacts, often resulting in patient disability and placing a strain on both families and communities. In 2019, IS-related fatalities totaled 3.29 million, representing half of stroke-related deaths and 17.7% of all cardiovascular disease fatalities. This underscores the critical significance of preventing IS [1].

This cerebrovascular event occurs due to the blockage or narrowing of arteries supplying blood to the brain. According to the TOAST (Trial of Org 101072 in Acute Stroke Treatment) classification, the most common cause is the formation of a blood clot within a blood vessel in the brain or neck, termed a thrombotic stroke. Another type, embolic stroke, occurs when a clot or other debris forms elsewhere in the body (often the heart) and travels to the brain, blocking blood flow [2].

Recent advances in stroke pathophysiology emphasize the significant role of inflammation and immunity in both stroke-related damage and mechanisms of neuroprotection and repair [3]. Few studies investigate the association of eosinophils and brain infarction in acute ischemic stroke (AIS) without hypereosinophilic syndrome. Other concurrent studies also attempted to elucidate the relationship between stroke and other blood cells such as erythrocytes, monocytes, and lymphocytes [4,5].

The management of AIS prioritizes the restoration of blood flow to the affected cerebral region and the mitigation of further neurological damage. Depending on individual clinical presentations, therapeutic interventions may encompass the administration of thrombolytic agents like alteplase, mechanical thrombectomy procedures, anticoagulant pharmacotherapy, and neuroprotective strategies. These modalities aim to facilitate recovery and facilitate the restoration of compromised neurological functions [6,7].

Recent studies endeavored to illuminate the unclarified aspect of eosinophils in AIS. However, the mechanisms underlying this role are poorly understood. This article delves into the intricate facets of ischemic stroke, exploring its relationship with hemoglobin, eosinophil count, and their association with thrombolytic treatment.

## Materials and methods

Study design and setting

The study was conducted in accordance with the Declaration of Helsinki and approved by the Medical Research Scientific and Ethical Evaluation Board of Kanuni Sultan Süleyman Training and Research Hospital (Approval No.: KAEK/2022.05.113; dated: 26/05/2022). From January 1, 2019, to April 1, 2022, all patients aged 18 years and over who were diagnosed with acute ischemic stroke in the emergency department of Kanuni Sultan Süleyman Training and Research Hospital and treated with intravenous recombinant tissue plasminogen activator (r-tPA) within 4.5 hours of stroke onset were included in this cross-sectional retrospective study. Patients were excluded if they met any of the following criteria: (1) evidence of acute infection at admission or any infection that occurred during the first 24 hours after admission; (2) diagnosed pro-inflammatory disease (malignancy, chronic inflammatory disease, chronic infectious disease, autoimmune disease) or immune suppressive state (immunosuppressive drug use or immune deficiencies); (3) acute myocardial infarction at admission or during the first 48 hours after admission; (4) transient ischemic attack less than 24 hours prior to admission; (5) previous stroke with partial recovery; (6) endovascular therapy or carotid endarterectomy following alteplase administration.

Medical history was obtained immediately upon hospital arrival. Subsequently, patients’ vital signs (body temperature, blood pressure, oxygen saturation, pulse, etc.) as well as finger stick blood glucose levels were measured and physical examination and 12-lead ECG were performed.

Neutrophil-to-lymphocyte ratio (NLR), platelet-to-lymphocyte ratio (PLR), and eosinophil-to-neutrophil ratio (ENR) were calculated. Gender, age, onset of symptoms, complaints, National Institutes of Health Stroke Scale (NIHSS) score, and stroke-affected area were recorded.

All individuals manifesting symptoms within 4.5 hours of onset and deemed eligible for intravenous thrombolysis (IVT) underwent non-contrast head computed tomography (CT) scans. Subsequent to the exclusion of intracranial hemorrhage and intracranial mass lesion, these patients underwent diffusion magnetic resonance (MR) scans and received intravenous alteplase therapy. Treatment with IVT was performed in accordance with the American Heart Association ischemic stroke guidelines [6]. The total dose of alteplase was 0.9 mg/kg up to a maximum dose of 90 mg: 10% of the total dose was administered intravenously within one minute, followed by an infusion of the remaining dose over 60 minutes. Patients were followed during treatment for any complications. Patients were divided into two groups: survivor and mortality groups.

Statistical analysis

Categorical data were presented as numbers and percentages. Statistical analyses were performed using SPSS software package version 25.0 (IBM Corp., Armonk, NY). For testing the normality of numerical variables, Kolmogorov-Smirnov and Shapiro-Wilk tests were used, along with skewness and kurtosis values. Data conforming to normal distribution were presented as mean and standard deviation, while non-conforming data were presented as median and interquartile range. Chi-square and Fisher's exact tests were used for the analysis of categorical variables, and Mann-Whitney U and Kruskal-Wallis tests were used for the comparison of continuous variables. Spearman test was conducted for correlation analysis of variables, and regression analysis was performed to determine the predictive effect on mortality estimation. All statistical analyses were considered significant at a 95% confidence interval, and p-values less than 0.05 were considered statistically significant.

## Results

A total of 61 people, including 33 men and 28 women, were included in the study. Four patients died during follow-ups. We observed mortality in three (9%) out of 33 male patients and in one (4%) out of 28 female patients. However, we did not find a significant relationship between gender and mortality (p = 0.371). Comparison of clinical characteristics between groups is shown in Table 1.

**Table 1:** Comparison of clinical features between survival and mortality groups. P < 0.05, Fisher’s exact test. ACA: anterior cerebral artery; MCA: middle cerebral artery; PCA: posterior cerebral artery.

Variables			Survival (57), n (%)	Mortality (4), n (%)	P-value
Gender	Male		30 (52.6)	3 (75)	0.371
	Female		27 (47.4)	1 (25)	
Plegia			26 (45.6)	4 (100)	0.53
Ataxia			6 (10.5)	0 (0)	0.654
Paresthesia			14 (24.6)	1 (25)	0.687
Dysarthria			21 (36.8)	0 (0)	0.175
Aphasia			8 (14)	0 (0)	0.561
Vertigo			5 (8.8)	0 (0)	0.704
Change in consciousness			8 (14)	0 (0)	0.561
MCA			43 (75.4)	3 (75)	0.687
ACA			6 (10.5)	0 (0)	0.65
PCA			10 (17.5)	1 (25)	0.559
Vertebrobasilar system			13 (22.8)	0 (0)	0.373

The mean duration of symptoms upon admission was 86.23 ± 56.37 minutes. The mean NIHSS score of patients was found to be 9.16 ± 3.88 (minimum: 4, maximum: 18). The median hemoglobin count was 13.9 in the survival group while 11.1 x 10³/µL in the mortality group (p = 0.014). No significant differences were found between the survival and mortality groups in terms of patients' symptoms, duration of symptoms, sites of involvement, and other laboratory parameters except for hemoglobin (Table 2).

**Table 2:** Comparison of clinical and laboratory variables between survival and mortality groups. P < 0.05, Mann-Whitney U test. The variables are presented as median (min-max). Reference range: WBC = 3.8-10 x 10³/µL; neutrophil = 1.78-5.38 x 10³/µL; platelets = 100-400 x 10³/µL; eosinophil = 0.04-0.54 x 10³/µL; lymphocyte = 0.8-4 x 10³/µL; hemoglobin = 13.0-17.5 g/dL. NIHSS: National Institutes of Health Stroke Scale; NLR: neutrophil-to-lymphocyte ratio; PLR: platelet-to-lymphocyte ratio; ENR: eosinophil-to-neutrophil ratio.

Variables		Survival (n = 57)	Mortality (n = 4)	P-value
Age (years)		64 (33-94)	78.5 (44-85)	0.255
Symptom onset time (minutes)		60 (15-240)	60 (30-120)	0.603
NIHSS		8 (4-18)	9.5 (6-12)	0.781
WBC count (x 10³/µL)		8.88 (2.76-29.58)	8.04 (5.72-9.33)	0.256
Neutrophil count (x 10³/µL)		5.8 (1.7-28.34)	5.01 (3.69-7.03)	0.431
Platelet count (x 10³/µL)		256 (100-421)	250 (168-298)	0.988
Eosinophil count (x 10³/µL)		0.09 (0.0-0.95)	0.15 (0.13-0.27)	0.189
Lymphocyte count (x 10³/µL)		1.8 (0.2-7.0)	1.75 (1.4-2.3)	0.896
Hemoglobin count (g/dL)		13.9 (9.3-17.2)	11.1 (10.6-11.9)	0.014
NLR		3.3 (0.62-56.68)	2.52 (2.13-5.02)	0.580
PLR		134.44 (34.71-398)	140.14 (80-206.43)	1.000
ENR		0.012 (0.0-0.27)	0.03 (0.02-0.06)	0.190

The correlation of mortality, NIHSS score, and symptom duration with age, WBC count, neutrophil, platelet, eosinophil, lymphocyte, hemoglobin count, NLR, PLR, and ENR has been analyzed. There was a statistically significant positive correlation between age and symptom duration (p < 0.002, r: 0.391). A statistically significant negative correlation was found between eosinophil count and NIHSS (p < 0.012, r: -0.321) and between eosinophil count and symptom duration (p < 0.042, r: -0.261). There was a negative correlation between hemoglobin levels and mortality (p < 0.013, r: -0.318). A statistically significant negative correlation was observed between ENR and NIHSS (p < 0.017, r: -0.305) as well as between ENR and symptom duration (p < 0.034, r: -0.271) (Table 3).

**Table 3:** The correlation between mortality, NIHSS, symptom duration, and continuous variables. P < 0.05, r: correlation coefficient; Spearman correlation analysis. NIHSS: National Institutes of Health Stroke Scale; WBC: white blood cells; Neu: neutrophil; Plt: platelets; Eos: eosinophil; Lymph: lymphocyte; Hgb: hemoglobin; NLR: neutrophil-to-lymphocyte ratio; PLR: platelet-to-lymphocyte ratio; ENR: eosinophil-to-neutrophil ratio.

Clinic features		Age	Symptom duration	NIHSS	Mortality	WBC	Neu	Plt	Eos	Lymph	Hgb	NLR	PLR	ENR
Mortality	r	0.147	-0.067	0.036	1.000	-0.147	-0.102	-0.002	0.169	-0.009	-0.318	-0.71	0.0	0.169
	p	0.259	0.608	0.783		0.259	0.436	0.989	0.192	0.943	0.013	0.584	1.00	0.192
NIHSS	r	-0.15	0.066	1.000	0.036	0.011	0.066	0.151	-0.321	-0.051	-0.227	0.064	0.121	-0.305
	P	0.911	0.611		0.783	0.936	0.616	0.244	0.012	0.697	0.079	0.625	0.352	0.017
Symptom duration	r	0.391	1.000	0.066	-0.067	-0.21	0.008	-0.077	-0.261	-0.116	-0.162	0.069	0.076	-0.271
	p	0.002		0.611	0.608	0.874	0.954	0.555	0.042	0.373	0.213	0.598	0.561	0.034

Hemoglobin is a significant predictor of mortality in the logistic regression model (p < 0.05, CI: 0.253-0.942). For each one-unit increase in hemoglobin, the odds of mortality decrease by a factor of 0.488 (Table 4).

**Table 4:** Regression analysis of hemoglobin value for mortality. B: coefficient.

Variable	B	P-value	OR	95% CI	
				Lower limit	Upper limit
Hemoglobin	-0.718	0.032	0.488	0.253	0.942

## Discussion

This study showed that lower eosinophil count is associated with higher NIHSS scores, which is relevant to more severe stroke (p < 0.012, r: -0.321). Moreover, a statistically significant negative correlation was found between eosinophil count symptom duration (p < 0.042, r: -0.261). Consistent with previous studies, no significant association was found between platelet count and mortality in this study. A contributing factor to the severity of stroke was shown as hemoglobin level in this study (p < 0.013, r: -0.318).

The central nervous system and the immune system are two powerful networks that work closely together [8]. Soon after ischemic stroke occurs, microglia and astrocytes are activated by local cytokines and chemokines. Inducement of these cells leads to the release of proinflammatory cytokines and free oxygen species [9]. Moreover, during an ischemic stroke, the integrity of the blood-brain barrier (BBB) is compromised. This disruption permits immune cells from the bloodstream to enter the ischemic brain. Consequently, these infiltrating immune cells, along with the damaged brain cells, release inflammatory mediators. These mediators induce brain edema or directly contribute to the death of brain cells in the penumbra, thereby causing further progression of infarct lesions. Peripheral immune changes triggered by stroke are believed to contribute to the pathogenesis of adverse complications such as secondary tissue damage, hemorrhagic transformation, and post-stroke infection [10]. To prevent such complications, it is necessary to have a better grasp of knowledge about pathophysiological events that occur in secondary damage, which helps to inhibit factors by "immunomodulation."

In response to an AIS, neutrophils act as the immune system's first responders. These white blood cells rapidly ramp up their activity and migrate to the injured brain tissue [11]. Infiltration of neutrophils into the ischemic core and surrounding penumbra following stroke contributes to a multifaceted neuroinflammatory response. Higher levels of neutrophils are associated with worse outcomes, including increased stroke severity, larger infarct volume, poorer recovery, and increased risks of new stroke [5,12]. In this study, the neutrophil count was not statistically significantly different between the survival and mortality groups. The apparent contradiction with the literature could be due to the insufficient number of participants in our study. New avenues for stroke treatment are emerging, thanks to research on neutrophils. Studies exploring ways to reduce circulating neutrophils, prevent their accumulation in the brain, and dampen their inflammatory activity show promise in lessening tissue death and improving patient recovery. These approaches hold potential as future immunotherapies for AIS [13].

Previous research indicates a negative association between eosinophil count and stroke severity, mortality risk, and poor functional outcomes in patients with AIS [12]. Several previous studies have attempted to elucidate the effects of higher eosinophil count in AIS through various mechanisms. These mechanisms can be broadly categorized into two main themes: lack of pro-inflammatory effects and anti-inflammatory effects of eosinophils. The release of inflammatory cytokines following cerebral ischemia causes eosinophils to migrate to the site of inflammation, leading to a reduction in peripheral blood eosinophils [13]. Thus, the higher eosinophil number is associated with less pro-inflammatory effect, better outcome, and smaller infarct size. As for another explanation based on the anti-inflammatory effect of eosinophil, elevated eosinophil levels promote enhanced eosinophil infiltration into ischemic tissues, bolstering their protective role. Therefore, individuals with low eosinophil counts, or eosinopenia, exhibit diminished neuroprotection and are more likely to experience adverse outcomes [12]. In contrast to previous studies, our investigation failed to identify a significant association between mortality and eosinophil count. This is likely attributable to the limited number of patients included in the mortality group.

NIHSS serves as a crucial tool for predicting the severity of acute cerebral infarction [14]. Our study revealed a statistically significant negative correlation between eosinophil count and NIHSS score. Wang et al., in their study involving patients with ischemic stroke, regardless of thrombolytic treatment status, identified a significant association between NIHSS and eosinophil count and ratio [12]. Intriguingly, our study revealed a significant difference in eosinophil levels between patients with varying symptom durations. Upon reviewing the existing literature, no previous study has reported a significant negative correlation between the time from symptom onset to hospital admission and eosinophil levels.

In the meta-analysis of Sadeghi et al., platelet count was found significantly low in AIS patients [15]. However, Lim et al. found that there was no significant association between platelet count and stroke severity [16]. Similarly, in this study, there was no association between mortality and platelet count. Our findings are consistent with those of Ghodsi et al., who investigated the association between platelet count and three-month and 12-month morbidity and mortality but did not find any significant relationship [17].

A decreased lymphocyte count has been shown to indicate severe brain injury and predicts poor neurological recovery within the first week, and unfavorable functional outcomes after three months [18]. Lymphocytes might play a protective role in the same way as eosinophils after AIS [19,20]. In this study, although no statistically significant relationship was found between lymphocyte count and mortality or NIHSS score, a negative correlation was observed in the correlation analysis. This is likely due to the small number of patients in the mortality group. This negative relationship is also supported by previous studies [21].

Due to the limitations of using a single inflammatory cell count to predict the prognosis of AIS, researchers are now exploring novel indicators by integrating different white blood cell subtypes, like NLR, PLR, and ENR, which can be easily performed in routine practice. Hori et al. found that the concurrent presence of neutrophilia and eosinopenia showed a significantly higher mortality rate than isolated neutrophilia [22]. However, the presence of neutrophilia without eosinopenia showed no significant difference in comparison to the absence of neutrophilia. Therefore, the prognostic significance of neutrophilia during cerebral infarction may only be evident when it is accompanied by eosinopenia. Considering all of these findings, it has been observed that the ratio of eosinophil to neutrophil counts is more effective in determining prognosis than the eosinophil and neutrophil levels alone [23].

In this study, a significant correlation was found between the ENR ratio and the NIHSS score, which indicates stroke severity and prognosis (p < 0.017). Besides, a negative correlation was found between the ENR and symptom duration in this study. It is known that the prognosis is worse in patients with longer time intervals between symptom onset and hospital admission [24]. In this study, both eosinophil count and ENR indirectly indicate that the prognosis is worse in patients with longer symptom duration.

While Qun et al. [25] and Zhao et al. [26] found an association between NLR and stroke prognosis, multivariate analysis in a study by Güneş showed that NLR had no prognostic value on its own [23]. Similarly, in our study, we did not find a significant correlation between NLR and mortality or NIHSS.

While elevated PLR is known to be associated with a worse prognosis, we did not find a significant correlation between PLR and mortality in this study, possibly due to the low number of patients in the mortality group [27]. Considering the findings of studies of Ghodsi et al. and Lim et al., the elevated PLR observed in patients with poor prognosis is not likely attributable to a concurrent rise in platelet count and decline in lymphocyte count, but rather to an isolated decrease in lymphocyte count in patients with poor prognosis [16,17].

Low hemoglobin levels increase the incidence of stroke [28]. In addition, a negative correlation was found between infarct size and hemoglobin level [29]. A significant association with mortality was also found in another study [30]. In our study, we also found a direct correlation between low hemoglobin levels and mortality (p < 0.013, r: -0.318).

It is important to acknowledge certain limitations in our study. The retrospective nature of our study, the small size of the mortality group, and the limited follow-up periods for patients can be considered as limitations of our study. Furthermore, comparing the discharge neurological statuses and blood parameters of surviving patients could have made the study more intriguing. However, we were unable to access relevant data.

## Conclusions

This study demonstrates the relationship between certain blood cell types and determining stroke prognosis. It has revealed the need for a detailed pathophysiological explanation of the role of leukocytes such as neutrophils, eosinophils, and lymphocytes in AIS. Based on our findings, we believe exploring the role of eosinophils, neutrophils, and lymphocytes in stroke-related immunomodulation would pave the way for potential newer modalities of treatment.

## References

[1] Benjamin EJ, Muntner P, Alonso A (2019). Heart disease and stroke statistics-2019 update: a report from the American Heart Association. Circulation.

[2] Adams HP Jr, Bendixen BH, Kappelle LJ, Biller J, Love BB, Gordon DL, Marsh EE 3rd (1993). Classification of subtype of acute ischemic stroke. Definitions for use in a multicenter clinical trial. TOAST. Trial of Org 10172 in Acute Stroke Treatment. Stroke.

[3] Kamel H, Iadecola C (2012). Brain-immune interactions and ischemic stroke: clinical implications. Arch Neurol.

[4] Semerano A, Strambo D, Martino G, Comi G, Filippi M, Roveri L, Bacigaluppi M (2020). Leukocyte counts and ratios are predictive of stroke outcome and hemorrhagic complications independently of infections. Front Neurol.

[5] Kim J, Song TJ, Park JH (2012). Different prognostic value of white blood cell subtypes in patients with acute cerebral infarction. Atherosclerosis.

[6] Powers WJ, Rabinstein AA, Ackerson T (2019). Guidelines for the early management of patients with acute ischemic stroke: 2019 update to the 2018 guidelines for the early management of acute ischemic stroke: a guideline for healthcare professionals from the American Heart Association/American Stroke Association. Stroke.

[7] Hart RG, Pearce LA, Aguilar MI (2007). Meta-analysis: antithrombotic therapy to prevent stroke in patients who have nonvalvular atrial fibrillation. Ann Intern Med.

[8] Elenkov IJ, Wilder RL, Chrousos GP, Vizi ES (2000). The sympathetic nerve—an integrative interface between two supersystems: the brain and the immune system. Pharmacol Rev.

[9] Jayaraj RL, Azimullah S, Beiram R, Jalal FY, Rosenberg GA (2019). Neuroinflammation: friend and foe for ischemic stroke. J Neuroinflammation.

[10] Shichita T, Ago T, Kamouchi M, Kitazono T, Yoshimura A, Ooboshi H (2012). Novel therapeutic strategies targeting innate immune responses and early inflammation after stroke. J Neurochem.

[11] Jickling GC, Liu D, Ander BP, Stamova B, Zhan X, Sharp FR (2015). Targeting neutrophils in ischemic stroke: translational insights from experimental studies. J Cereb Blood Flow Metab.

[12] Wang A, Quan K, Tian X (2021). Leukocyte subtypes and adverse clinical outcomes in patients with acute ischemic cerebrovascular events. Ann Transl Med.

[13] Ruhnau J, Schulze J, Dressel A, Vogelgesang A (2017). Thrombosis, neuroinflammation, and poststroke infection: the multifaceted role of neutrophils in stroke. J Immunol Res.

[14] Agis D, Goggins MB, Oishi K (2016). Picturing the size and site of stroke with an expanded National Institutes of Health Stroke Scale. Stroke.

[15] Sadeghi F, Kovács S, Zsóri KS, Csiki Z, Bereczky Z, Shemirani AH (2020). Platelet count and mean volume in acute stroke: a systematic review and meta-analysis. Platelets.

[16] Lim HH, Jeong IH, An GD (2019). Early prediction of severity in acute ischemic stroke and transient ischemic attack using platelet parameters and neutrophil-to-lymphocyte ratio. J Clin Lab Anal.

[17] Ghodsi H, Abouei Mehrizi MA, Khoshdel AR, Shekarchi B (2021). Evaluation of combining Alberta Stroke Program Early CT Score (ASPECTS) with mean platelet volume, plateletcrit, and platelet count in predicting short- and long-term prognosis of patients with acute ischemic stroke. Clin Neurol Neurosurg.

[18] Urra X, Cervera A, Villamor N, Planas AM, Chamorro A (2009). Harms and benefits of lymphocyte subpopulations in patients with acute stroke. Neuroscience.

[19] Liesz A, Hu X, Kleinschnitz C, Offner H (2015). Functional role of regulatory lymphocytes in stroke: facts and controversies. Stroke.

[20] Park BJ, Shim JY, Lee HR (2011). Relationship of neutrophil-lymphocyte ratio with arterial stiffness and coronary calcium score. Clin Chim Acta.

[21] Zierath D, Olmstead T, Stults A, Shen A, Kunze A, Becker KJ (2018). Chemical sympathectomy, but not adrenergic blockade, improves stroke outcome. J Stroke Cerebrovasc Dis.

[22] Hori YS, Kodera S, Sato Y, Shiojiri T (2016). Eosinopenia as a predictive factor of the short-term risk of mortality and infection after acute cerebral infarction. J Stroke Cerebrovasc Dis.

[23] Güneş M (2020). Is neutrophil/eosinophil ratio at admission a prognostic marker for in-hospital mortality of acute ischemic stroke?. J Stroke Cerebrovasc Dis.

[24] Lee EJ, Kim SJ, Bae J (2021). Impact of onset-to-door time on outcomes and factors associated with late hospital arrival in patients with acute ischemic stroke. PLoS One.

[25] Qun S, Tang Y, Sun J (2017). Neutrophil-to-lymphocyte ratio predicts 3-month outcome of acute ischemic stroke. Neurotox Res.

[26] Zhao L, Dai Q, Chen X (2016). Neutrophil-to-lymphocyte ratio predicts length of stay and acute hospital cost in patients with acute ischemic stroke. J Stroke Cerebrovasc Dis.

[27] Tang Y, Xu H, Du X (2006). Gene expression in blood changes rapidly in neutrophils and monocytes after ischemic stroke in humans: a microarray study. J Cereb Blood Flow Metab.

[28] Panwar B, Judd SE, Warnock DG, McClellan WM, Booth JN 3rd, Muntner P, Gutiérrez OM (2016). Hemoglobin concentration and risk of incident stroke in community-living adults. Stroke.

[29] Bellwald S, Balasubramaniam R, Nagler M (2018). Association of anemia and hemoglobin decrease during acute stroke treatment with infarct growth and clinical outcome. PLoS One.

[30] Barlas RS, Honney K, Loke YK (2016). Impact of hemoglobin levels and anemia on mortality in acute stroke: analysis of UK regional registry data, systematic review, and meta-analysis. J Am Heart Assoc.

